# Comparative Evaluation of Machine Learning and Conventional Material Decomposition Algorithms for Spectral Chest Radiography Using a CdTe Photon-Counting Detector

**DOI:** 10.3390/s26103202

**Published:** 2026-05-19

**Authors:** Sriharsha Marupudi, Bahaa Ghammraoui

**Affiliations:** Division of Imaging, Diagnostics, and Software Reliability, Office of Science and Engineering Labs, U.S. Food and Drug Administration, Silver Spring, MD 20993, USA

**Keywords:** photon counting, chest radiography, material decomposition, Monte Carlo, low-contrast detectability, Poisson statistics, machine learning

## Abstract

Spectral chest radiography with photon-counting detectors (PCDs) enables energy-resolved acquisition for bone/soft-tissue separation, but quantitative performance depends on detector cross-talk and the selected material decomposition algorithm. We performed a controlled simulation study comparing a conventional low-order polynomial decomposition model with two machine learning regressors (multilayer perceptron (MLP) and support vector regression (SVR)) for a cadmium telluride (CdTe) PCD. A Geant4-derived detector response model, coupled with a charge-transport model, was integrated into a physics-forward model including charge sharing and Poisson quantum noise. Digital LucAl/IEC 62220-2-1 phantoms with aluminum and polymethyl methacrylate inserts were used for quantitative bias/root mean square error (RMSE) evaluation, and task-based low-contrast detectability that was evaluated using an exponential transformation of the free-response operating characteristic (EFROC) method using a matched-filter template. Performance was evaluated over clinically relevant dose levels (0.07–7.5 mAs), calibration grid densities (3×3 to 8×8), and numbers of energy thresholds (M=2–6). Polynomial decomposition was stable under sparse calibration, whereas ML methods benefited strongly from denser calibration and additional thresholds; SVR achieved the lowest RMSE under dense calibration, while MLP produced smoother maps and improved soft-tissue detectability at low-to-intermediate dose. At high dose, all methods approached near-ideal detection performance. These results quantify practical trade-offs between calibration requirements, quantitative accuracy, and low-contrast detectability for PCD-based spectral chest radiography.

## 1. Introduction

Spectral X-ray imaging using photon-counting detector (PCD) systems has emerged as an advancement over conventional energy-integrating detectors, due to improved quantitative accuracy, enhanced tissue contrast, and superior material discrimination [1,2,3]. By directly counting individual photons and resolving their energies into multiple bins, PCDs enable energy-resolved acquisition with higher spatial resolution and reduced electronic noise, making them well-suited to spectral computed tomography (CT), advanced radiographic imaging, and material decomposition [4]. Unfortunately, PCD measurements are affected by detector nonidealities such as charge sharing, pulse pileup, and cross-talk, which distort the measured spectra and can degrade quantitative spectral performance [1,4,5].

In chest radiography, spectral information is particularly useful because it enables separation of bone and soft-tissue equivalent attenuation, which can improve visualization and detection of low-contrast structures [1,4,5]. Standardized attenuation phantoms and insert geometries (e.g., LucAl and IEC 62220-2-1) also provide a controlled methodology for evaluating performance trade-offs in dose, contrast, and material separation [6,7,8].

Material decomposition aims to estimate basis material thicknesses from energy-dependent measurements by exploiting the energy dependence of X-ray interactions. This inverse problem is nonlinear and ill-posed: the relationship between detected photon counts and material thickness depends on the incident spectrum, object attenuation, detector spectral response, and energy threshold configuration [9,10,11]. At low dose, Poisson quantum noise and spectral distortions propagate through the inversion and can introduce increased variance and residual cross-talk between decomposed material images [9,10,11].

Physics-informed calibration approaches, including low-order polynomial models, provide a computationally efficient mapping between spectral measurements and basis thicknesses and can be robust when calibrated within clinically relevant ranges [9]. However, polynomial models may exhibit reduced generalization outside the calibration range and can become less stable when the measurement space is poorly conditioned (e.g., limited calibration sampling or reduced spectral sensitivity at higher energy thresholds). In contrast, machine learning (ML) regression methods can learn the nonlinear mapping directly from calibration data and have shown promising performance in photon-counting material decomposition studies [12,13]. ML performance, however, can depend strongly on training set size, sampling, the number of energy thresholds, and measurement noise levels.

While more recent deep learning methods such as convolutional neural networks (CNNs) and physics-informed neural networks (PINNs) have been developed for spectral CT material decomposition, the present study focuses on low-complexity regression models that simplify computational intensity and cost and enable a controlled comparison under consistent calibration conditions [14,15,16]. In this work, material decomposition is performed on a per-pixel basis using a low-dimensional feature, thus not requiring a spatial context. Additionally, the physics-based forward model provides a physically consistent training method without explicitly defining physics constraints in the learned model.

In this study, a comparison methodology was designed to evaluate representative regression strategies for pixel-wise material decomposition for low-dimensional spectral measurements under matched calibration and acquisition conditions. We selected a low-order polynomial model as the conventional baseline as polynomial calibration-based mappings are computationally efficient and are widely used in spectral material decomposition [17,18]. To represent ML methods we selected multilayer perceptron (MLP) regression and support vector regression (SVR), to provide two nonlinear regression methods: a parametric neural network model and a kernel-based method. Both are well-suited to the ill-posed material decomposition problem, with the input of a per-pixel feature vector of log-normalized energy bin measurements rather than large image patches. In contrast, more complex model architecture such as CNNs are more advantageous when spatial context is a primary component of the learning task. The aim of this work was to isolate the effect of each regression model class under a common physics-based forward model and calibration method, thus we focused on methods that operate directly on the spectral measurement vector and avoided additional confounding from spatially dependent image priors.

In this work, we present a controlled comparative evaluation of conventional and ML-based material decomposition methods for spectral chest radiography using a simulated cadmium telluride (CdTe) PCD system. A Geant4-derived detector response model is combined with a physics-forward model incorporating charge sharing and Poisson quantum noise. Using a modified LucAl phantom geometry (IEC 62220-2-1) with aluminum and polymethyl methacrylate (PMMA) representing bone- and soft-tissue-equivalent materials, we evaluate (i) a low-order polynomial decomposition model, (ii) MLP regression, and (iii) SVR. Performance is studied across clinically relevant dose levels, numbers of energy thresholds, and calibration grid sizes, using both quantitative accuracy root mean square error (RMSE) and task-based low-contrast detectability assessed via exponential transformation of the free-response operating characteristic (EFROC) analysis.

Task-based metrics such as EFROC quantify signal detectability for a specified imaging task (e.g., low-contrast object detection), which is closely related to clinically relevant diagnostic tasks. However, there is generally no universal threshold value of EFROC performance that defines diagnostic adequacy, as required detectability depends on the specific clinical task, lesion characteristics, and imaging conditions. Accordingly, EFROC is most appropriately interpreted in a relative sense, enabling comparison of imaging methods and acquisition conditions for a given task.

The results provide practical insight into the trade-offs between model complexity, calibration, and low-dose imaging performance in photon-counting spectral chest radiography.

### 1.1. Phantom Simulation and Image Acquisition Parameters

#### 1.1.1. Digital Phantom Design

A set of digital phantoms were generated to support both (i) quantitative material decomposition accuracy assessment and (ii) task-based low-contrast detectability (LCD) evaluation. The phantoms were based on the LucAl concept (Standard Dosimetric/Calibration Phantom; CDRH/FDA) and the IEC 62220-2-1 insert geometry, with minor modifications [6,7,8]. The LucAl phantom has been reported to exhibit good spectral equivalence to anthropomorphic chest phantoms, supporting its use for simulating clinically relevant radiographic conditions [7,19].

**Figure 1:**
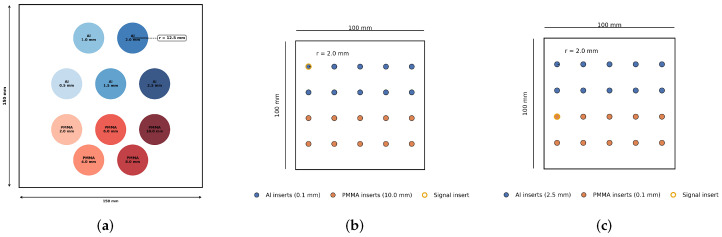
Digital phantoms used in this work. (**a**) Quantitative evaluation phantom based on the IEC 62220-2-1/LucAl concept with aluminum and PMMA inserts spanning clinically relevant thickness ranges. (**b**) Low-contrast detectability (LCD) phantom for aluminum detection: aluminum inserts are fixed at 0.1mm thickness while PMMA is fixed at 10.0mm. (**c**) Low-contrast detectability (LCD) phantom for PMMA detection: PMMA inserts are fixed at 0.1mm thickness while aluminum is fixed at 2.5mm. All phantoms were initially simulated on a 0.1mm micro-pixel grid and later rebinned to 1.0mm macro-pixels for analysis.

Quantitative material decomposition phantom: For quantitative evaluation, we used the modified IEC/LucAl insert plate shown in Figure 1a. The phantom spans 150×150mm in-plane and was generated on a micro-pixel grid with 0.1mm pitch to enable accurate modeling of material boundaries and detector-level effects (e.g., charge sharing). The uniform background consisted of 4.1mm Al and 83mm PMMA. Ten circular inserts (radius r=12.5mm) were superimposed and divided into two material groups. The Al inserts had additional thicknesses of 0.5, 1.0, 1.5, 2.0, and 2.5mm, while the PMMA inserts had additional thicknesses of 2.0, 4.0, 6.0, 8.0, and 10.0mm. The inserts were placed to avoid overlap and minimize edge interactions. Each insert locally increases only one basis-material thickness while leaving the other unchanged, thereby producing known combinations of Al and PMMA path lengths for evaluating material decomposition accuracy across a clinically relevant dynamic range.

Task-based LCD phantoms: For EFROC-based LCD evaluation (Figure 1b,c), two additional phantoms were generated, each with dimensions of 100×100mm and circular disk inserts of radius r=2.0mm. In the Al-LCD configuration (Figure 1b), Al inserts were fixed at 0.1mm thickness (signal-present task), while PMMA inserts were fixed at 10.0mm. In the PMMA-LCD configuration (Figure 1c), PMMA inserts were fixed at 0.1mm thickness, while Al inserts were fixed at 2.5mm.

#### 1.1.2. X-Ray Spectrum and Primary Transmission

The incident spectrum was generated using SpekPy for a tungsten anode operated at 120 kVp with a 12° anode angle and 2.0 mm Al filtration at a source-to-image distance (SID) of 1000 mm [20]. Energy-dependent attenuation coefficients were obtained from the National Institute of Standards and Technology (NIST) XCOM photon cross-section library (for Al and the constituent elements of PMMA), and linear attenuation coefficients were computed using material densities of $1.18\;g/{\mathrm{cm}}^{3}$ for PMMA and $2.699\;g/{\mathrm{cm}}^{3}$ for Al. For each micro-pixel, the polychromatic primary transmission spectrum was computed using Beer–Lambert attenuation (Equation (1)) under the two-material assumption

(1) $$I\left(E\right)=I_{0}\left(E\right)\exp\;\left[-\mu_{\mathrm{Al}}\left(E\right)\;x_{\mathrm{Al}}-\mu_{\mathrm{PMMA}}\left(E\right)\;x_{\mathrm{PMMA}}\right],$$

where $I_{0}\left(E\right)$ is the incident fluence spectrum, $\mu_{m}\left(E\right)$ is the linear attenuation coefficient of material *m*, and $x_{m}$ is the corresponding path length.

The transmitted spectrum was then linearly interpolated to the detector incident energy grid prior to application of the detector response model.

#### 1.1.3. CdTe PCD Response and Covariance Modeling

A two-step procedure was used to estimate the CdTe PCD response, including (i) the mean spectral response (DRF) and (ii) the spatio-energetic covariance structure induced by charge sharing and spectral redistribution (PcTK-style modeling) [21,22].

First, Geant4 Monte Carlo simulations were used to model photon interactions and energy deposition in the CdTe sensor for monoenergetic incident photons spanning 15–174 keV in 1 keV increments [23,24]. Energy-deposition events were recorded and mapped onto a local 3×3 pixel neighborhood using a native pixel pitch of 0.1mm (100 μm) to capture inter-pixel charge sharing and short-range spatial cross-talk with limited computational complexity [21]. A 0.1 mm pitch represents a small-pixel CdTe PCD configuration, where smaller anode geometries are commonly used to support high count-rate operation and to better resolve charge-sharing effects [1].

Second, a custom Python charge-transport and signal-formation model was applied to the Geant4 energy-deposition data. Charge sharing was modeled using a Gaussian charge-cloud formulation (drift/diffusion/repulsion), and spectral broadening was modeled by including electronic noise and Fano statistics, with additional terms to account for incomplete charge collection. From the event-level response, we computed (i) the mean detector response and (ii) the covariance of the measurement vector (e.g., energy-bin/threshold counts over the local pixel neighborhood), capturing inter-bin and inter-pixel correlations caused by spatio-energetic cross-talk [21,22].

**Figure 2:**
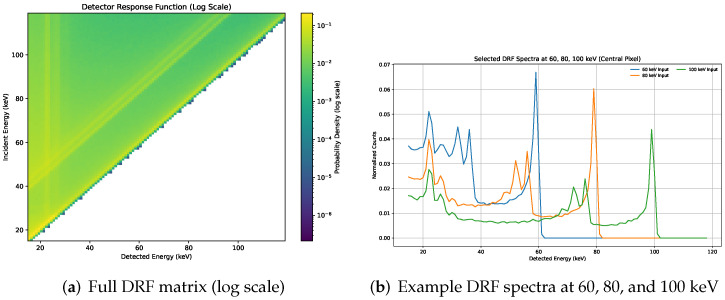
Example CdTe photon-counting detector response function (DRF) used in this work. (**a**) Full DRF matrix $R_{ij}$ (log scale), illustrating spectral redistribution from incident energy $E_{\mathrm{in}}$ to detected energy $E_{\mathrm{out}}$. (**b**) Example DRF spectra for 60, 80, and 100 keV incident photons (central pixel).

The mean response was summarized by a detector response function (DRF) matrix $R_{ij}$, where each row corresponds to an incident energy bin $E_{i}$ and each column corresponds to a detected energy bin *j*. The DRF is used in the forward model to map the transmitted spectrum to expected detected counts in each energy bin. Figure 2 shows an example DRF used in this work.

Pulse pileup effects were not modeled (i.e., a low-rate regime without pileup distortion was assumed) [1]. Scatter was also not included in the forward model; we assume scatter suppression and/or correction via an ideal anti-scatter grid and/or dedicated scatter estimation/removal methods (e.g., moving-block–based correction), as described in prior work [25].

Spatial charge sharing was approximated by a normalized 3×3 convolution kernel independently applied to each detected energy bin. The kernel was constructed using a Gaussian charge-cloud model and calibrated by fitting the Gaussian standard deviation to the Monte Carlo-derived DRF simulations. The resultant kernel was applied with a 2D convolution to each energy bin image. The linear convolutional approximation preserves the charge-sharing behavior and provides a computationally efficient model for the forward projection simulation.

Finally, after generating noisy energy-bin images at the native 0.1mm sampling, the data was rebinned by 10×10 summation to form 1.0mm macro-pixels for the material decomposition and task-based evaluations. This separates the detector-response characterization (performed at native pitch) from the subsequent image sampling used to match typical digital radiographic pixel size.

### 1.2. Material Decomposition Models

All material decomposition methods estimated aluminum and PMMA thickness maps from the same set of energy-resolved measurements and were trained/evaluated using identical calibration conditions to enable a consistent comparison. The input feature vector for each pixel consisted of the log-normalized energy-bin measurements as depicted in Equation (2),

(2) $$f_{k}=-\log\left(\frac{n_{k}}{n_{k,0}}\right),\;k=1,\ldots ,N_{b},$$

where $n_{k}$ denotes the measured photon counts in detected energy bin *k*, $n_{k,0}$ denotes the corresponding open-beam counts, and $N_{b}$ is the number of energy bins/thresholds. This log transform helps linearize the attenuation dependence and provides consistent scaling across energy channels [26,27].

Calibration grids were generated from noise-free forward-model outputs spanning clinically relevant Al and PMMA thickness ranges. Noise-free calibration was used to isolate model bias from stochastic variance and to ensure consistent training across all methods. This choice allows for differences in test performance to be attributed more directly to the decomposition method than to calibration noise realizations. Noisy calibration experiments were not included in this study as the primary objective was to isolate model class behavior under matched calibration conditions. However, calibration noise is expected to be an important practical factor in experimental systems and is considered further in the discussion.

After training, the models were applied to noisy measurements generated at multiple dose levels. Five independent noise realizations were generated per dose level using independent random seeds by sampling from a Poisson distribution with mean equal to the forward-projected expected detector counts.

The forward-projected expected counts include spectral redistribution through the DRF and spatial charge-sharing effects modeled via the convolution kernel described above. The subsequent Poisson sampling therefore represents an approximation in which charge-sharing effects are incorporated in the mean detector response, while the stochastic fluctuations are assumed to be independent across macro-pixels and energy bins. As a result, the full spatio-energetic covariance induced by charge sharing is not explicitly propagated in the noise generation.

Performance was evaluated as a function of: (i) calibration grid size (e.g., 3×3, 5×5, and 8×8 sampling of the Al/PMMA thickness domain), (ii) the number of energy thresholds/bins $N_{b}$, and (iii) photon fluence (dose). The evaluated photon counts were $1\times 10^{5}$, $2.3\times 10^{6}$, and $1\times 10^{7}$ photons, corresponding to 0.07, 1.70, and 7.50 mAs, respectively, which are representative of clinically relevant chest radiography dose levels [28]. Photon counts were converted to equivalent tube current–time products (mAs) using the incident spectrum and system geometry.

#### 1.2.1. Polynomial Regression Model

Material decomposition was performed using a second-degree polynomial regression model that maps the log-normalized measurement vector $f=[f_{1},\ldots ,f_{N_{b}}]$ to basis thickness estimates $\hat{t}=\left[{\hat{t}}_{\mathrm{Al}},{\hat{t}}_{\mathrm{PMMA}}\right]$ [9,13].

A third-degree polynomial model was also evaluated but yielded minimal improvement in material separation while increasing model complexity and was therefore not further pursued. The polynomial coefficients were fit using the noise-free calibration grid described above.

#### 1.2.2. MLP Regression

MLP was implemented to model the nonlinear mapping from the log-normalized measurements f to basis thicknesses. The MLP consisted of two hidden layers with 128 neurons each, ReLU activations, and a linear output layer producing ${\hat{t}}_{\mathrm{Al}}$ and ${\hat{t}}_{\mathrm{PMMA}}$. A dropout rate of 0.01 and L2 weight decay were applied for regularization. The network was trained using the Adam optimizer and a smooth L1 loss function to improve robustness to outliers while maintaining sensitivity to small residuals [29].

The MLP architecture and the hyperparameters were selected based on preliminary tuning and a lightweight architecture sensitivity analysis to balance model capacity and generalization with the nonlinear material decomposition performance. Networks with one to three hidden layers and 32, 64, 128, and 256 neurons per hidden layer were trained using the same noise-free calibration data as the polynomial model and validated on aluminum and PMMA thickness ranges. Smaller networks such as one-hidden-layer models showed limited ability to capture the nonlinear mapping between log-normalized spectral measurements and material thickness. Two-hidden-layer models had a substantial reduction in validation RMSE and reached a performance plateau. The selected model, consisting of two hidden layers with 128 neurons per layer, achieved validation RMSE of 0.033±0.005 mm for Al and 0.217±0.020 mm for PMMA, corresponding to a mean material RMSE of 0.125±0.012 mm. Larger networks did not display substantially superior performance with a small reduction in mean validation RMSE (0.120±0.057 mm). The results of this analysis are provided in Figure S1. The larger 256 neurons per layer required 3.9 times more trainable parameters and had greater variability across initialization seeds. The two-hidden-layer 128-neuron architecture was chosen as a stable compromise between accuracy, parameter efficiency, and generalization. The regularization parameters such as the dropout and L2 weight decay were added to the model to promote smoothness and robustness to noise in the material map images.

#### 1.2.3. SVR

SVR was implemented as a nonlinear baseline method offering greater flexibility than polynomial regression while remaining more constrained than the MLP model. The SVR hyperparameters were selected based on preliminary tuning to provide a flexible but stable nonlinear baseline for the material decomposition task, balancing model smoothness and robustness to noise. A radial basis function (RBF) kernel was used to introduce nonlinear feature interactions while limiting overfitting [30,31]. The regularization parameter *C* controls the trade-off between fitting the calibration data and maintaining smooth generalization; the selected value avoided both excessive smoothing and overfitting. The parameter ϵ defines the width of the insensitive-loss region and therefore affects robustness to small fluctuations; the chosen value suppressed fitting to minor fluctuations while preserving sensitivity to meaningful variations in material thickness. The final SVR hyperparameters used in this study were C=100, ϵ=0.010, and gamma set to scale. These values provided stable performance across the evaluated calibration densities and dose levels without substantial overfitting.

Training was also performed on a noise-free calibration grid, and the trained SVR was applied to noisy data across the evaluated dose range.

(3) $$\hat{y}\left(x\right)=\sum_{i=1}^{N}\left(\alpha_{i}-\alpha_{i}^{*}\right)K\left(x_{i},x\right)+b,$$

where *x* is the feature vector (here, x=f), $x_{i}$ denotes the *i*th support vector, $\alpha_{i}$ and $\alpha_{i}^{*}$ are the learned dual coefficients, K(·,·) is the kernel function, *b* is the bias term, and $\hat{y}\left(x\right)$ represents the estimated material thickness.

Tree-based regressors (e.g., decision trees and random forests) were considered but not pursued due to their sensitivity to noise and limited smooth extrapolation behavior for continuous regression tasks. All methods were trained and evaluated using noise-free calibration data, with identical input features and acquisition conditions.

### 1.3. Performance Evaluation

#### Quantitative Accuracy: Bias and RMSE

The quantitative performance of the material decomposition methods was assessed using bias and RMSE, consistent with our prior work [2]. Metrics were computed separately for each material (Al, PMMA) and for each known insert thickness (five thickness levels per material). For a given thickness level, the estimated thickness was measured as the mean value within a circular ROI centered in the corresponding insert location (excluding the insert boundary to reduce partial-volume effects) and aggregated across five independent Poisson noise realizations. Let *N* denote the number of realizations (ROI measurements) for a given material and thickness level.

- Bias (systematic error) (Δ):
  (4) $$\Delta =\frac{1}{N}\sum_{i=1}^{N}\left({\hat{t}}_{i}-t^{true}\right),$$
  where ${\hat{t}}_{i}$ is the ROI-averaged estimated thickness for realization *i* and $t^{true}$ is the corresponding ground-truth insert thickness.
- RMSE:
  (5) $$\mathrm{RMSE}=\sqrt{\frac{1}{N}\sum_{i=1}^{N}{\left({\hat{t}}_{i}-t^{true}\right)}^{2}}.$$
  RMSE summarizes the combined effects of systematic error and variability across realizations.

### 1.4. Signal Detection and Low-Contrast Detectability

Task-based image quality was evaluated using an exponential free-response receiver operating characteristic (EFROC) method with unknown-location signal searching [32]. Two 100×100mm digital phantoms were used (Figure 1b,c), each containing circular disk inserts of radius r=2.0mm. For the *Al-detection* task, aluminum signal inserts were fixed at 0.1mm thickness and the non-target PMMA thickness was fixed at 10.0mm. For the *PMMA-detection* task, PMMA signal inserts were fixed at 0.1mm thickness and the non-target aluminum thickness was fixed at 2.5mm. The thick non-target material was selected to create a challenging detection task because residual material cross-talk and structured decomposition artifacts are typically strongest in high-attenuation regions.

For each dose level, R=10 independent noise realizations were generated, producing paired signal-present and signal-absent material images with identical acquisition parameters. A calibration grid size of 8×8 was fixed along with *M* = 4 energy thresholds. Signal-absent images were generated by removing a single target disk (Al for the Al-detection task; PMMA for the PMMA-detection task), while keeping all other inserts unchanged.

Signal searching was performed on each material image using a matched-filter observer approach (Equation (6)) with a circular disk template matched to the known signal radius. The discrete template was defined as a binary disk normalized to unit sum,

(6) $$h\left(r\right)=\left\{\begin{array}{cc} \frac{1}{\left|\Omega \right|}, & ∥r∥\;\leq r,\\ 0, & \mathrm{otherwise}, \end{array}\right.$$

where Ω denotes the set of pixels within the disk support. This normalization yields a response proportional to the local mean within the disk and provides consistent scaling across dose levels. The spatial response map was computed using fast Fourier convolution.

For each signal-present image, the true-positive decision variable was defined as the maximum matched-filter response within an acceptance region centered at the known target location. False-positive marks were obtained from the corresponding signal-absent images by identifying local maxima that exceeded the high percentile threshold of the response distribution. False-positive searching was performed over (i) a uniform background region and (ii) a region containing the non-target (thick) material inserts (PMMA regions when evaluating Al images; Al regions when evaluating PMMA images) to account for both random noise and structured residual artifacts.

The EFROC performance metric, AFE, was calculated as the area under the transformed EFROC curve using the nonparametric EFROC estimator [32]:

(7) $${\hat{A}}_{FE}=\frac{1}{I}\sum_{i=1}^{I}\exp\;\left[-\frac{1}{N}\sum_{j=1}^{J}H\left(y_{j}-x_{i}\right)\right],$$

where $\left\{x_{i}\right\}$ are the true-positive scores (from signal-present images), $\left\{y_{j}\right\}$ are false-positive scores (from signal-absent images), *N* is the number of signal-absent images, and H(z) is where

$$H\left(z\right)=\left\{\begin{array}{cc} 1 & \mathrm{if}\;z>0,\\ \frac{1}{2} & \mathrm{if}\;z=0,\\ 0 & \mathrm{if}\;z<0. \end{array}\right.$$

Error bars were computed using the Hanley–McNeil formula for area under the curve (AUC) variance [33], as depicted in Equation (8).

(8) $$\sigma_{\mathrm{AUC}}=\sqrt{\frac{AUC\left(1-AUC\right)+\left(N_{t}-1\right)\;\left(\frac{AUC}{2-AUC}-AUC^{2}\right)+\left(N_{f}-1\right)\;\left(\frac{2AUC^{2}}{1+AUC}-AUC^{2}\right)}{N_{t}N_{f}}}$$

where $N_{t}$ and $N_{f}$ are the positive and negative scores from the signal searching algorithm.

## 2. Results and Discussion

### 2.1. Qualitative Material Decomposition Maps

**Figure 3:**
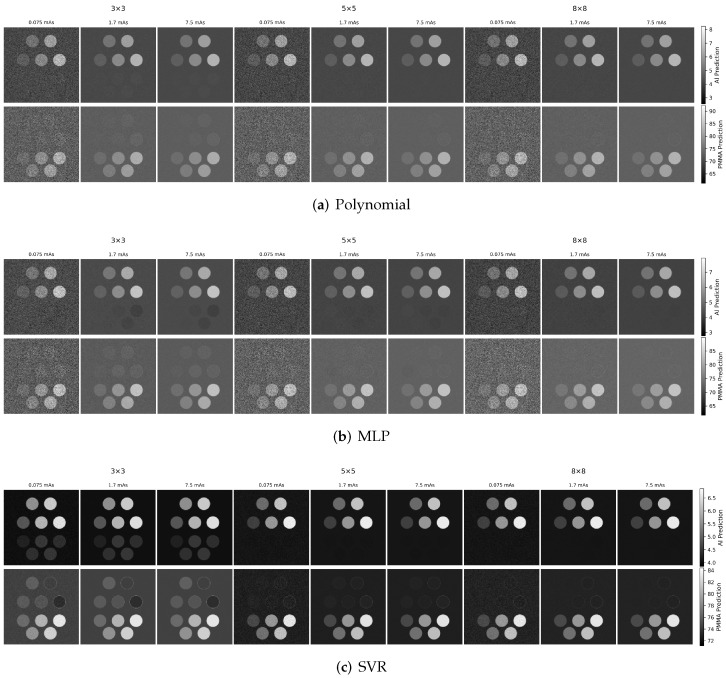
Representative aluminum and PMMA material maps for the evaluated decomposition methods, shown for three dose levels and three calibration grid sizes (3×3, 5×5, and 8×8) using two energy thresholds. The top row shows aluminum thickness maps and the bottom row shows PMMA thickness maps. Increasing dose reduces image noise for all methods, while larger calibration grids reduce residual cross-talk, with the strongest calibration-grid dependence observed for the machine learning models.

Representative aluminum and PMMA material maps for the evaluated decomposition methods are shown in Figure 3. Results are shown for three dose levels and three calibration grid sizes (3×3, 5×5, and 8×8) using two energy thresholds. For each method, the top row shows the estimated aluminum thickness map and the bottom row shows the estimated PMMA thickness map. From visual inspection, several consistent trends are observed. First, the overall noise level decreases with increasing dose for all methods, most clearly in the uniform background regions and in the lower-contrast inserts. At the highest dose level, material boundaries appear more uniform and better defined across all calibration grid sizes.

Second, the calibration grid size has a strong impact on the machine learning models. Both MLP and SVR show improved material separation and reduced residual cross-talk as the calibration grid increases from 3×3 to 8×8. In contrast, the polynomial model exhibits relatively consistent performance across the evaluated grid sizes, indicating greater robustness to sparse calibration sampling.

Third, residual structures and imperfect material separation are most prominent in the SVR results, while the polynomial results generally show less residual structure artifacts. This behavior is due to the local fitting nature of the RBF kernel. Although SVR mapping is smooth in the feature space, each prediction is dominated by nearby support vectors in the log-normalized spectral measurement space. In chest radiography, the wide aluminum and PMMA thickness ranges include highly attenuating conditions where spectral measurements become compressed and are less sensitive to additional changes in thickness. Due to the local ill conditioning, small measurement variations or sparsely sampled calibration can change the relative influence of nearby support vectors, resulting in structured residual cross-talk in the decomposed images. The SVR material maps appear particularly sensitive to calibration grid size with reduced cross-talk at larger calibration grid size. Changes in the dose are less visually pronounced for SVR under some conditions, suggesting model performance is more limited by calibration density and model bias than by Poisson quantum noise.

**Figure 4:**
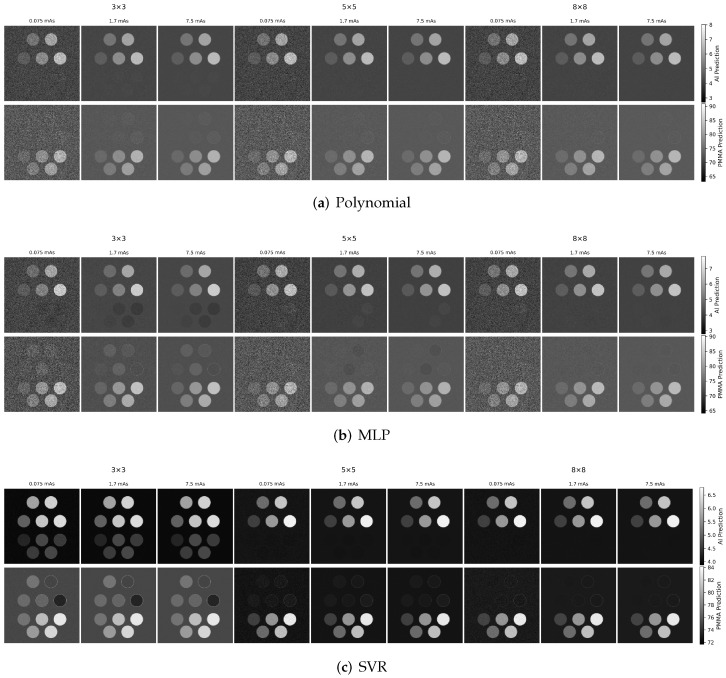
Representative aluminum and PMMA material maps for the evaluated decomposition methods using six energy thresholds. Results are shown for the same phantom and calibration grid sizes (3×3, 5×5, and 8×8). Compared with the two-threshold case (Figure 3), increased spectral sampling provides a modest reduction in residual cross-talk, most apparent for the MLP and SVR results. The top row shows aluminum thickness maps and the bottom row shows PMMA thickness maps.

To illustrate the impact of increased spectral sampling, Figure 4 shows the corresponding material maps using six energy thresholds. Compared with the two-threshold case, increasing the number of thresholds produces a modest improvement in material discrimination, most noticeably for the MLP and SVR models in terms of reduced residual cross-talk. Overall, however, the dominant qualitative trends with dose and calibration grid size remain similar, indicating that both spectral sampling and calibration density contribute to performance, with calibration density being a key factor for the machine learning models.

Noise-free calibration was used in this study as a controlled approximation to isolate model bias from stochastic variability in the calibration process and to ensure matched training conditions across all decomposition methods. This choice allows for differences in test performance to be attributed more directly to the decomposition model class rather than to calibration-noise realizations. In practical systems, however, calibration measurements are themselves noisy and may also be affected by phantom uncertainty, spectral drift, threshold or gain instability, and residual mismatch between the physical system and the forward model. Such errors can propagate through the learned inverse mapping as both systematic bias and increased variance in the decomposed material estimates. Under these more realistic conditions, lower-order parametric models would be expected to be more robust to noisy or sparse calibration data. Lower-order parametric models contain fewer fitted parameters and impose a smoother global mapping, whereas more flexible models such as MLP and SVR may better represent nonlinear detector response but may also be more sensitive to calibration noise unless additional regularization, repeated calibration measurements, or averaging strategies are used. This interpretation is qualitatively consistent with the present results, in which the polynomial model remained comparatively stable under sparse calibration, whereas the MLP and SVR benefited more strongly from denser calibration grids.

### 2.2. Quantitative Accuracy: Bias and RMSE

**Figure 5:**
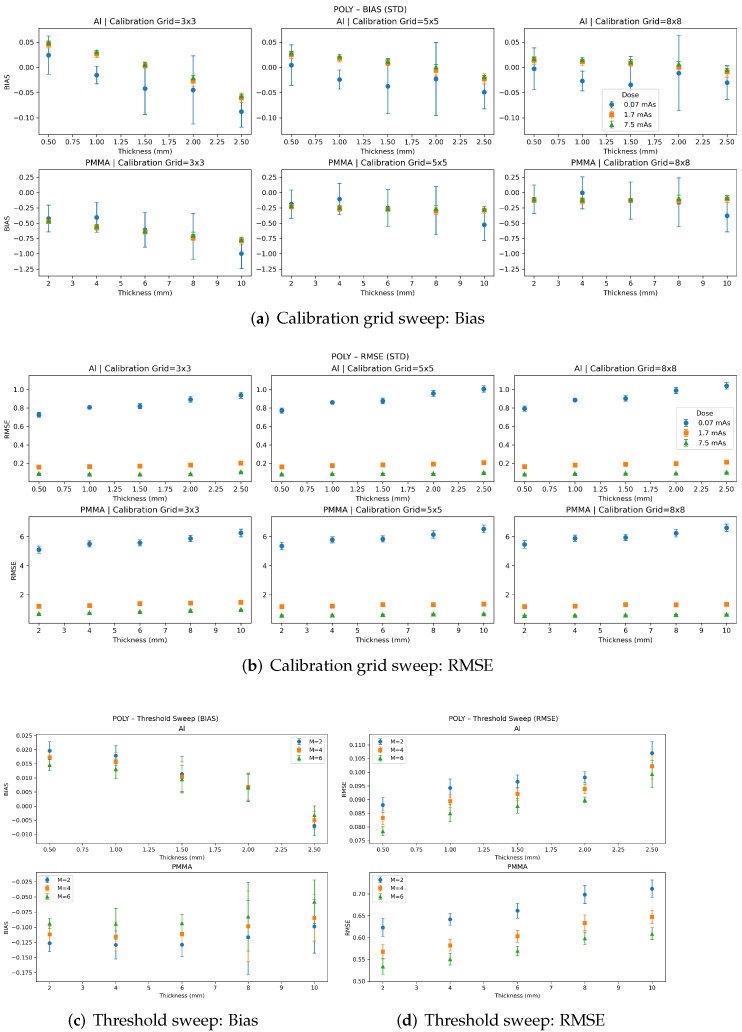
Performance of the polynomial-based material decomposition as a function of calibration-grid density and number of energy thresholds. Only modest changes in bias and RMSE can be observed with increasing calibration grid size, and increasing dose reduces RMSE. Monotonic improvement can be observed with increasing energy thresholds. Error bars denote the standard deviation across five independent noise realizations. For certain data points, the error bars are difficult to discern because of their small magnitude.

**Figure 6:**
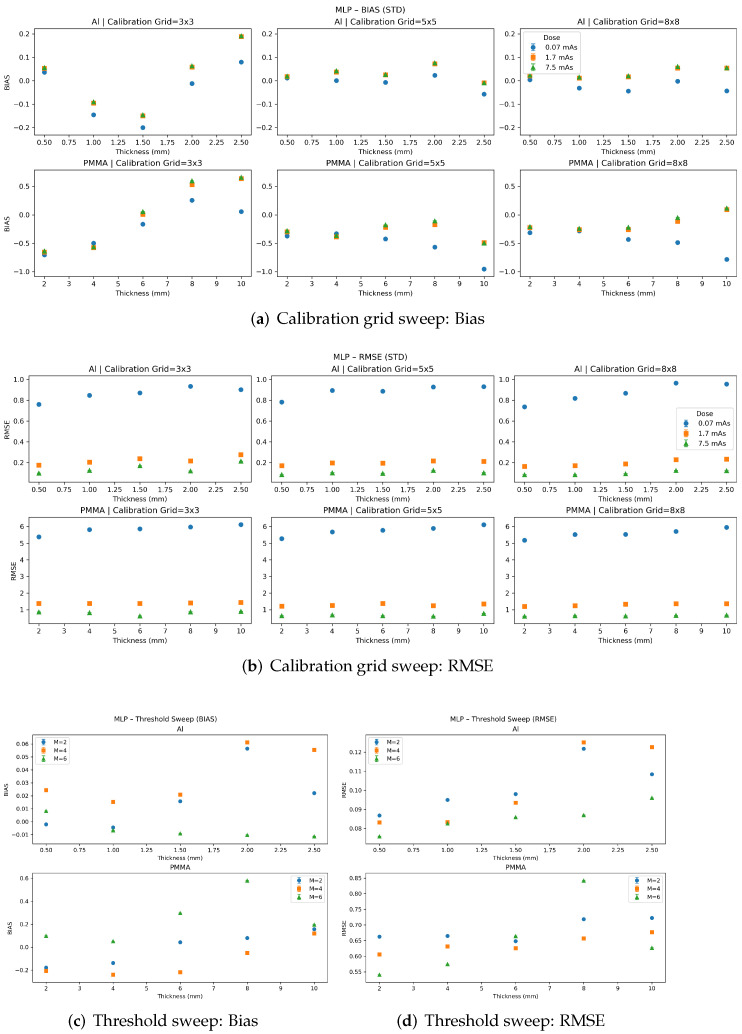
Performance of the MLP-based material decomposition as a function of calibration-grid density and number of energy thresholds. Increasing calibration grid size substantially reduces RMSE and bias. The model is less monotonic with increasing energy thresholds, suggesting sensitivity to the balance between added spectral information and noise propagation/model regularization. Error bars denote the standard deviation across five independent noise realizations. For certain data points, the error bars are difficult to discern because of their small magnitude.

**Figure 7:**
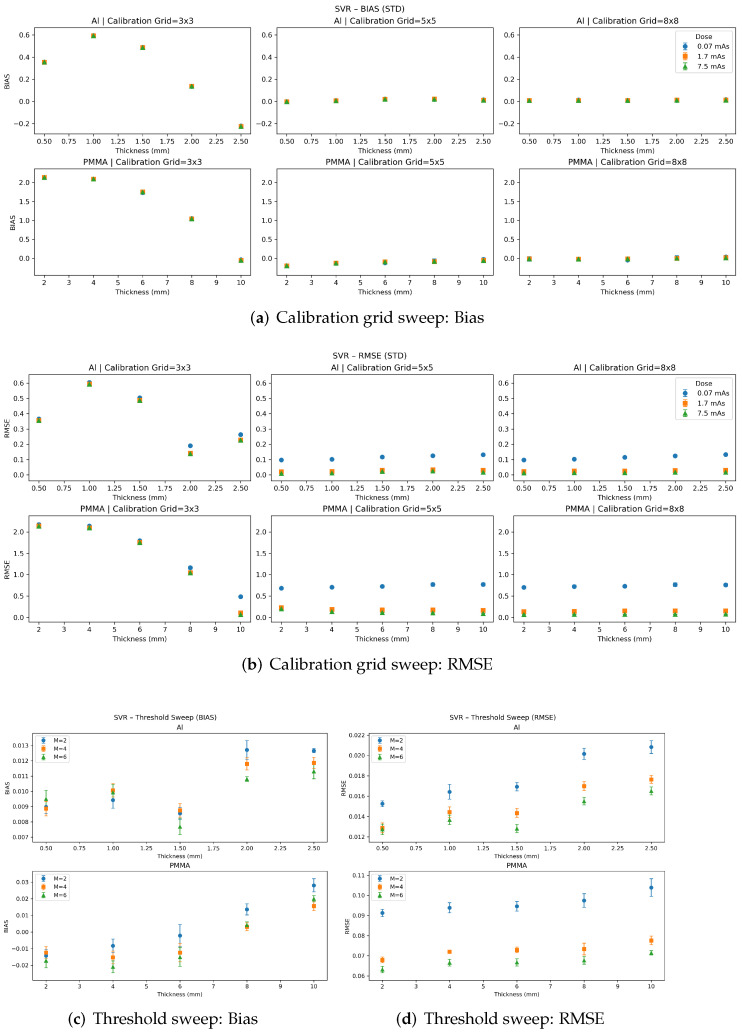
Performance of the SVR-based material decomposition as a function of calibration-grid density and number of energy thresholds. Increasing calibration grid size greatly improves model performance with a strong reduction in RMSE and bias. The model is monotonic to increasing energy thresholds, with greater quantitative performance at a higher number of thresholds. Error bars denote the standard deviation across five independent noise realizations. For certain data points, the error bars are difficult to discern because of their small magnitude.

Figure 5, Figure 6 and Figure 7 summarize the per-insert quantitative accuracy of the polynomial, MLP, and SVR material decomposition methods in terms of bias and RMSE. Results are reported separately for each insert thickness for both materials. For each method, panels (a,b) show the calibration-grid sweep (RMSE and bias, respectively) across dose levels for three calibration grid sizes 3×3 samples, 5×5 samples, and 8×8 samples. Panels (c,d) show the threshold sweep (RMSE and bias, respectively) for M=2,4,6 energy thresholds, evaluated at a fixed dose of 7.5 mAs and a fixed calibration grid size of 8×8. Error bars denote the standard deviation across five independent noise realizations. For certain data points, the error bars are difficult to discern because of their small magnitude.

For the polynomial model (Figure 5), the calibration-grid sweep results depicted in (a,b) show relatively stable performance as the calibration grid density increases from 3×3 to 8×8, with only modest changes in bias and RMSE. This indicates that the low-order polynomial mapping remains well-constrained even with limited calibration sampling. Dose has a clear effect, with reduced variability and lower RMSE at higher mAs.

In contrast, the MLP and SVR methods (Figure 6 and Figure 7) show stronger dependence on calibration-grid density. For the sparsest calibration grid (3×3), both methods exhibit increased error, with the SVR showing the most pronounced degradation in both bias and RMSE. Increasing the calibration density to 5×5 and 8×8 substantially improves performance. MLP and SVR achieve very low RMSE, with minimal bias among the evaluated methods once sufficient calibration sampling is used.

Photon-counting systems require dense sampling of the material thickness domain to capture the nonlinear detector response and as a result are very time-consuming. In this work the calibration grid sizes (3×3) and (8×8) represent more sparse sampling compared to typical experimental calibration measurements. The results indicate the ML methods are achieving strong performance at moderately dense sampling. Furthermore, training is performed offline, while inference is computationally efficient and operates on a per-pixel basis with low complexity. This makes the proposed approaches feasible for practical imaging applications.

The threshold-sweep results (panels (c,d)) show that increasing the number of energy thresholds generally improves quantitative performance, with the most consistent monotonic improvement observed for the polynomial and SVR models. For the MLP model, performance is less monotonic across energy thresholds, suggesting sensitivity to the balance between added spectral information and noise propagation/model regularization. Overall, these results reinforce the trade-off between model complexity and calibration requirements: polynomial decomposition provides robust performance with limited calibration data, while ML-based methods have greater performance with increased sampling and, in many cases, with increased spectral sampling.

### 2.3. Task-Based Low-Contrast Detectability (EFROC)

**Figure 8:**
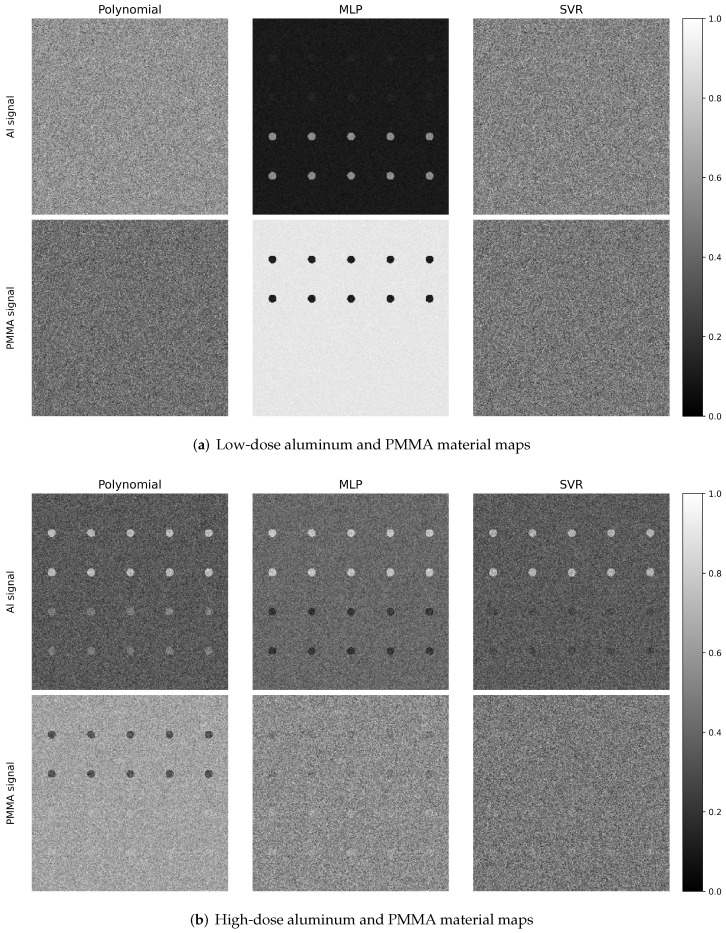
Comparison of polynomial, MLP, and SVR material maps for the PMMA detection task phantom at low dose. The two rows show reconstructed Al and PMMA map signals for the two digital phantoms. Material maps show cross-talk and structured decomposition artifacts from highly attenuating opposing-material inserts.

Figure 8a,b depict representative signal-present material maps for the aluminum- and PMMA-signal phantoms, respectively. For both signal configurations, residual cross-talk is visible in the opposing material channel. Consistent with the material maps shown in Figure 3, the results demonstrate that highly attenuating opposing-material inserts exhibit residual material cross-talk and structured decomposition artifacts. This incomplete cancelation introduces background texture, thereby reducing signal conspicuity and increasing the difficulty of the detection task.

**Figure 9:**
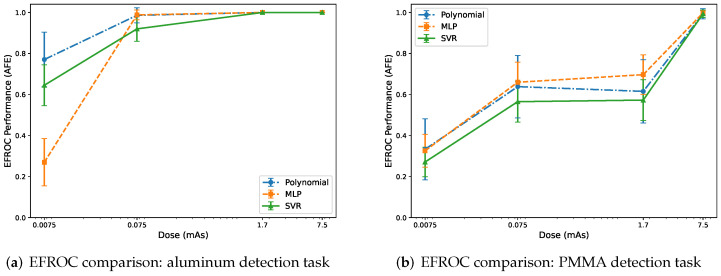
EFROC signal-detection performance (AFE) as a function of dose for (**a**) aluminum and (**b**) PMMA insert detection using polynomial, MLP, and SVR material decomposition. Results are shown for a fixed calibration grid size of (8×8) samples and M=4 energy thresholds. Detection performance improves with increasing dose due to reduced Poisson noise and improved material separation, and all approaches studied converged toward near-ideal performance at the highest dose in (**a**). For the PMMA detection task in (**b**), MLP has higher AFE than the polynomial and SVR model at low and intermediate dose. All models converge towards near-ideal performance at high dose. Error bars represent one standard deviation and were computed using Equation (8).

Figure 9 summarizes task-based signal-detection performance using the EFROC metric, AFE, for aluminum and PMMA insert detection. Results are shown as a function of dose for a fixed calibration grid size of (8×8) samples and M=4 energy thresholds.

Overall, detectability improves with increasing dose for both aluminum and PMMA detection tasks, consistent with reduced quantum noise and improved contrast in the decomposed material maps. For aluminum detection (Figure 9a), the polynomial method exhibits the highest AFE at the ultra-low dose point, while the MLP shows the most reduced performance at this extreme noise level. At 0.075 mAs and above, all methods improve substantially and rapidly approach near-ideal detection performance (AFE ≈1), indicating that material decomposition is no longer the limiting factor at moderate-to-high dose.

For PMMA detection (Figure 9b), performance increases more gradually with dose, reflecting the lower intrinsic contrast of the PMMA task and stronger sensitivity to residual decomposition cross-talk. At low and intermediate dose levels, the MLP achieves slightly higher AFE than the polynomial and SVR methods, while SVR generally shows the lowest detectability. At the highest dose (7.5 mAs), all methods converge toward near-ideal performance.

These results indicate that low-contrast detectability is governed by the combined effects of quantum noise and residual material cross-talk: ML-based decomposition can provide improved soft-tissue (PMMA) detectability in the low-to-intermediate dose regime with sufficient calibration and spectral sampling, while the polynomial approach remains robust and reaches near-ideal performance once dose is sufficiently high.

There is no universal threshold value of EFROC performance that defines diagnostic adequacy, as required performance depends on the specific task, lesion characteristics, and imaging conditions. EFROC values are interpreted as values approaching 1.0 corresponding to near-certain detection and lower values indicating increasing detection difficulty. Prior studies have demonstrated that intermediate values (0.70–0.90) representing meaningful differences in detection performance. In this study, all methods approach AFE≈1 at higher dose levels, indicating near-ideal detectability. Differences observed at low and intermediate dose levels therefore reflect practically relevant variations in detection performance under dose-limited conditions.

### 2.4. Count Rate Limitations and Pulse Pileup Considerations

Pulse pileup effects were not modeled in the present detector simulation, and the results therefore represent a low-count-rate regime without dead-time-related count loss or pileup-induced spectral distortion. Pulse pileup becomes significant when the incident count rate approaches the inverse of the detector dead time (τ). Standard detector models show that deviations from linear counting begin when Rτ≳0.1, with severe count losses and spectral distortion occurring as Rτ→1[1]. For CdTe PCDs, maximum count-rate capabilities are on the order of $10^{7}$–$10^{8}$ counts/s/pixel, with practical operating ranges for minimal pileup effects typically in the $10^{6}$–$10^{7}$ counts/s/pixel regime [1,4,5].

Although this work evaluated low-dose chest radiography conditions, pulse pileup is affected by local instantaneous count rate rather than by dose alone. Therefore, high-fluence regions in clinical chest radiography, where the count rate is higher in direct beam areas such as the lung region, may experience stronger pileup effects than represented in the simulation. Pulse pileup can result in count loss and spectral distortion through temporal overlap of photon events, mixing counts between high and low energy bins, and deviations in the log-normalized measurements used for material decomposition. The quantitative bias, RMSE, and EFROC trends reported here should be interpreted as performance under low pulse pileup conditions and should not be extrapolated directly to high-count-rate detector operation without additional modeling.

The impact of pulse pileup-induced spectral distortion may also depend on the decomposition method. The polynomial model relies on a fixed low-order calibration mapping and would be expected to develop bias if the detector response under clinical high-count-rate conditions differs from the calibration response. However, because the polynomial model is relatively constrained, it may be less sensitive to small deviations in the distribution than more flexible regression models.

In contrast, the MLP and SVR models can learn nonlinear mappings and may better accommodate pileup-induced spectral distortion if such effects are included in the training or calibration data. However, when trained only on pileup-free data, these ML models may be more sensitive to mismatch between training and testing conditions. Under high-count-rate conditions, particularly in lung or direct-beam regions, pileup could move the measured spectral features outside the learned training domain, and the ML models could therefore deteriorate earlier than the polynomial model despite their greater flexibility under matched conditions.

### 2.5. Residual Scatter and Model Robustness

Scatter effects were not included in the forward model; therefore, the results represent an idealized condition in which scatter is assumed to be fully suppressed or corrected. However, in clinical chest radiography, residual scatter may remain after correction and can be a major source of structured artifacts in material-decomposed images. Residual scatter can introduce an additive, spatially varying, and energy-dependent bias in the measured energy-bin counts, which can alter the log-normalized input features used for material decomposition. As a result, residual material cross-talk or background texture may be introduced into the material-decomposed images.

A residual scatter component of approximately 5–10% could challenge the robustness of the material decomposition methods evaluated in this work if the calibration or training data are scatter-free. The polynomial model relies on a fixed low-order calibration mapping and would likely develop bias when applied to measurements containing residual scatter if such effects were not modeled in the calibration data. However, because the polynomial model is relatively constrained and smooth, its degradation may be more gradual for small distribution shifts. In contrast, the MLP and SVR methods represent more complex nonlinear mappings. As a result of this flexibility, their performance depends more strongly on the training-data distribution. If residual scatter is not included during training, the resulting scatter-contaminated measurements may fall outside the learned calibration manifold.

This issue may be particularly relevant for SVR. With an RBF kernel, the SVR prediction depends on the similarity between the test input feature vector and the support vectors learned from the calibration data. A spatially varying residual scatter component can shift the measurements away from the scatter-free support-vector distribution, potentially leading to larger bias and structured artifacts.

Thus, under mismatched conditions, a 5–10% residual scatter component may challenge SVR robustness to a greater extent than polynomial decomposition, despite the strong quantitative performance of SVR under the scatter-free conditions evaluated in this work. However, if scatter effects are included in the calibration or training data, ML models may be able to learn the scatter-contaminated mapping and recover improved robustness.

### 2.6. Relation to Prior PCD Material Decomposition Studies

The findings in this work should also be interpreted in relation to recent work on material decomposition with PCDs. Prior studies have explored neural-network-based material decomposition under pulse-pileup conditions [13], deep learning approaches for multi-material spectral CT [34], and physics-informed neural network estimators [16]. Although these studies provide relevant context, direct numerical comparison with this work is challenging due to the difference in detector assumptions, target materials, calibration strategy, and evaluation metrics. The controlled study complements methods used in the broader literature isolating how conventional and ML regression models behave under clinically relevant spectral chest radiography conditions.

## 3. Conclusions

This work presented a controlled comparison of conventional polynomial and machine learning material decomposition methods for photon-counting spectral chest radiography using a simulated CdTe PCD with a Geant4-derived detector response and a physics-forward model including charge sharing and Poisson quantum noise. Using IEC/LucAl-inspired phantoms with aluminum and PMMA basis materials, performance was evaluated across clinically relevant dose levels, calibration grid densities, and numbers of energy thresholds, using both per-insert quantitative metrics (bias/RMSE) and task-based low-contrast detectability (EFROC).

Several trends in the model were observed. First, the low-order polynomial model showed comparatively stable behavior as calibration sampling was reduced, indicating robustness to sparse calibration grids. Second, the performance of ML-based methods (MLP and SVR) benefited strongly from increased calibration density, with SVR achieving the best quantitative accuracy once sufficient calibration sampling was available, while the MLP produced smoother material maps and improved soft-tissue (PMMA) detectability in the low-to-intermediate dose regime. Third, increasing the number of energy thresholds generally improved quantitative performance with diminishing returns, and the ML methods showed greater sensitivity to spectral sampling and calibration density than the polynomial model. Finally, for moderate-to-high dose conditions, all approaches converged toward near-ideal detection performance, indicating that algorithm choice is most critical for low-dose conditions or sparsely calibrated grids.

This study has several limitations. Scatter and pulse pileup were not modeled, and performance was evaluated in simulation under idealized assumptions for scatter suppression/correction. As discussed above, pulse pileup may be particularly relevant in high-local-fluence regions of clinical chest radiography, such as direct-beam or low-attenuation lung regions, and may affect polynomial and ML decomposition methods differently depending on whether pileup effects are represented in the calibration or training data. Consequently, the reported bias, RMSE, and EFROC trends should be interpreted as applying to a controlled, low-pileup simulation rather than to all clinical count-rate conditions.

In clinical chest radiography, complex anatomical structures, scatter effects, threshold/gain instability, and count-rate-dependent pulse pileup may introduce additional spectral distortions not represented in the present training or testing data. Therefore, the conclusions of this study should be interpreted as preliminary findings that require confirmation under clinically relevant acquisition conditions.

From a clinical perspective, the structured residuals observed for SVR may limit its use as a stand-alone decomposition method under sparse calibration, low-dose, or highly attenuating chest-imaging conditions. These residual artifacts could obscure low-contrast soft-tissue regions. More dense calibration, optimized energy threshold selection, and additional regularization or spatial consistency constraints may reduce these artifacts and should be evaluated in future experimental studies.

Future work will address these limitations by incorporating realistic scatter modeling and higher-count-rate detector effects, including dead-time and pulse-pileup effects, to assess algorithm robustness under conditions closer to clinical chest radiography. Additional work will investigate threshold optimization and calibration strategies under spectral distortion and will validate these findings using measurements with physical phantoms and more anatomically realistic phantoms.

## Data Availability

The data presented in this study are available on request from the corresponding author.

## Supplementary Materials

The following supporting information can be downloaded at https://www.mdpi.com/article/10.3390/s26103202/s1, Figure S1: Validation RMSE for Al, PMMA, and mean material-thickness estimation as a function of the number of neurons per hidden layer for MLPs with 1, 2, and 3 hidden layers. Each model was trained using the same noise-free slab-calibration framework and evaluated on an independent dense slab-validation grid spanning the Al/PMMA thickness range used in the main study. Error bars denote the standard deviation across three random initialization seeds. Validation error decreased substantially when moving from one to two hidden layers, while additional width or depth produced diminishing returns. The selected two-hidden-layer, 128-neuron architecture was near the validation-RMSE plateau and provided a stable compromise between accuracy and parameter efficiency.
