# Supplementary figures and images for: Comparative Evaluation of Machine Learning and Conventional Material Decomposition Algorithms for Spectral Chest Radiography Using a CdTe Photon-Counting Detector
Source: Sensors (Basel). 2026 May 19;26(10):3202. doi: 10.3390/s26103202 (PMC13210418; doi:10.3390/s26103202)

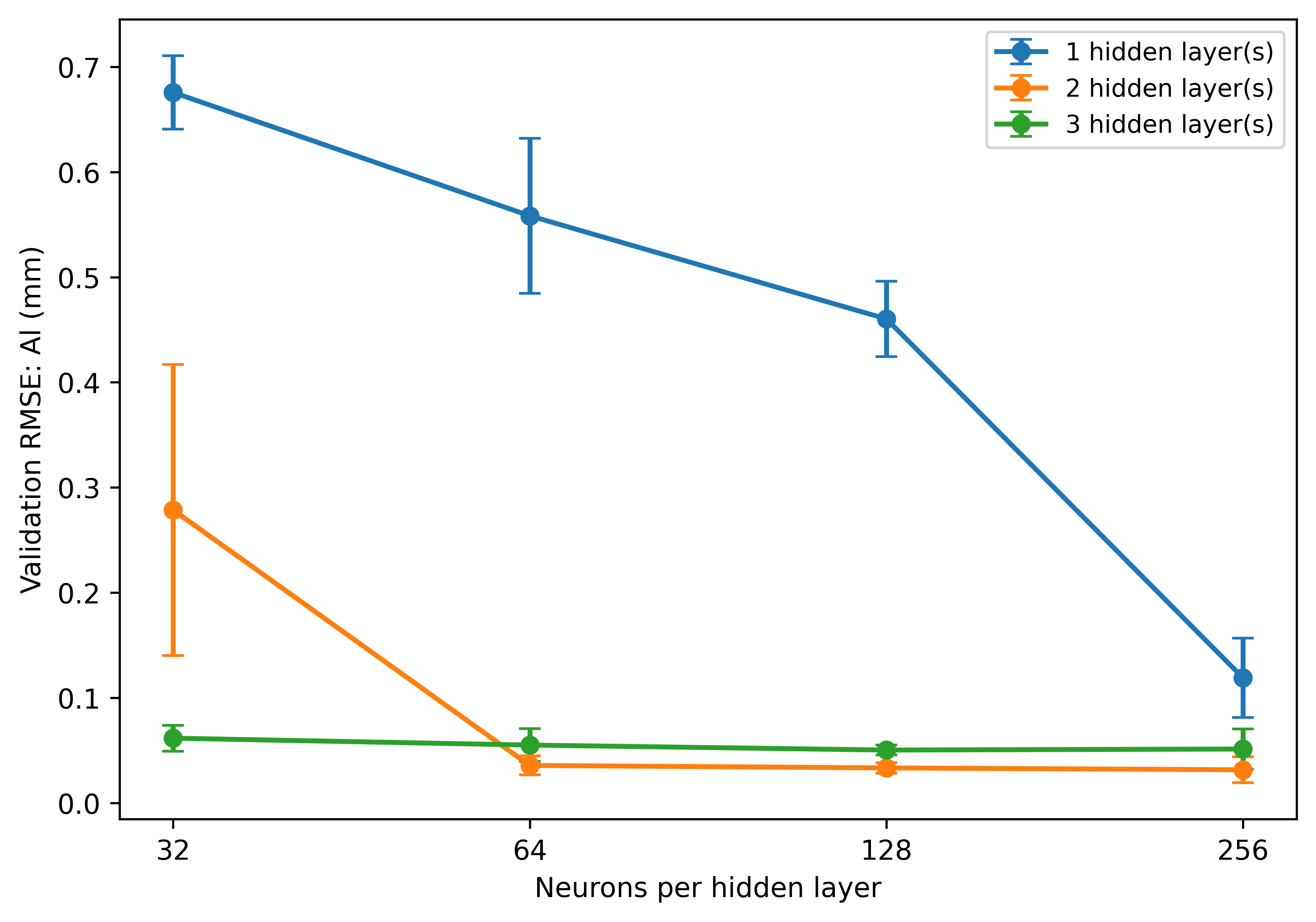

Supplement: Supplementary file 1 [file sensors-26-03202-s001.zip › sensors-4260470-mlp_architecture_ablation_al_rmse.png]

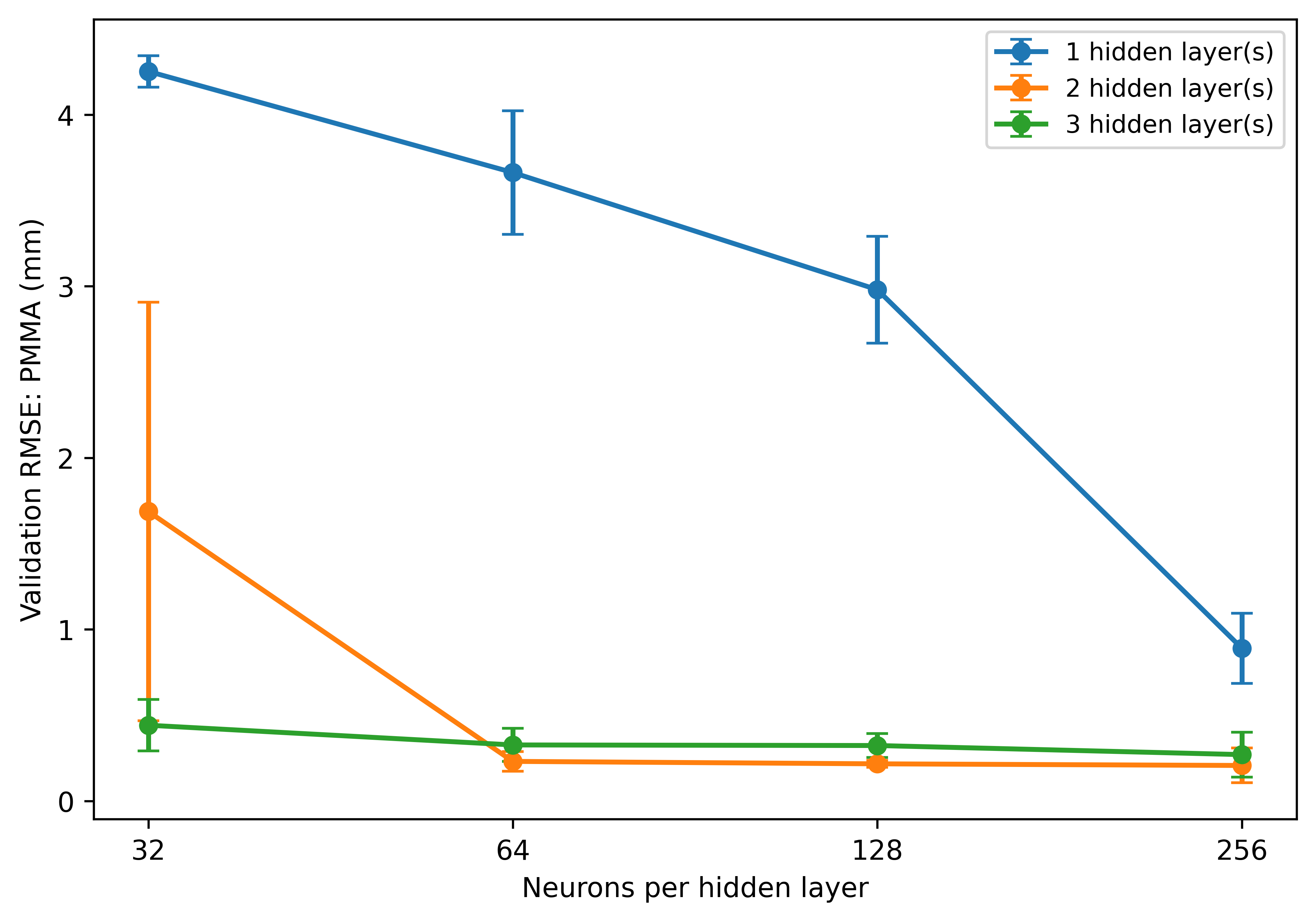

Supplement: Supplementary file 1 [file sensors-26-03202-s001.zip › sensors-4260470-mlp_architecture_ablation_pmma_rmse.png]
